# Human rights and the sexual and reproductive health of women living with HIV – a literature review

**DOI:** 10.7448/IAS.18.6.20290

**Published:** 2015-12-01

**Authors:** Shubha Kumar, Sofia Gruskin, Rajat Khosla, Manjulaa Narasimhan

**Affiliations:** 1 Department of Preventive Medicine, Institute for Global Health, University of Southern California, Los Angeles, CA, USA; 2 Program on Global Health & Human Rights, Institute for Global Health, University of Southern California, Los Angeles, CA, USA; 3 Department of Reproductive Health & Research, World Health Organization, Geneva, Switzerland

**Keywords:** human rights, sexual health, reproductive health, women living with HIV, healthcare

## Abstract

**Introduction:**

Even as the number of women living with HIV around the globe continues to grow, realization of their sexual and reproductive health and human rights remains compromised. The objective of this study was to review the current state of knowledge on the sexual and reproductive health and human rights of women living with HIV to assess evidence and gaps.

**Methods:**

Relevant databases were searched for peer-reviewed and grey literature. Search terms included a combination of MeSH terms and keywords representing women, HIV/AIDS, ART, human rights, sexual and reproductive health. We included both qualitative and quantitative literature published in English, French, or Spanish between July 2011 and December 2014.

**Results and discussion:**

The search yielded 2228 peer-reviewed articles, of which 40 met the inclusion criteria in the final review. The grey literature search yielded 2186 documents of which seven met the inclusion criteria in the final review. Of the articles and documents reviewed, not a single peer-reviewed article described the explicit implementation of rights in programming, and only two documents from the grey literature did so. With one possible exception, no articles or documents were found which addressed rights comprehensively, or addressed the majority of relevant rights (i.e. equality; non-discrimination; participation; privacy and confidentiality; informed decision making; availability, accessibility, acceptability and quality (3AQ) of services individually or in their totality; and accountability). Additional findings indicate that the language of rights is used most often to describe the apparent neglect or violation of human rights and what does exist only addresses a few rights in the context of a few areas within sexual and reproductive health.

**Conclusions:**

Findings from this review suggest the need to better integrate rights into interventions, particularly with attention to provider training, service delivery, raising awareness and capacity building among the community of women living with HIV. Further research is urgently needed to support the sexual and reproductive health and rights of women living with HIV, to identify what works and to inform future programming and policies to improve care, treatment and support for women living with HIV.

## Introduction

Realization of the sexual and reproductive health (SRH) of women living with HIV remains a key challenge, in part due to a lack of integration of human rights in health programming and policies affecting them, and often because of the outright neglect and violation of their human rights in many aspects of their lives. Reproductive health can be understood as a state of complete physical, mental and social well-being, and not merely the absence of disease or infirmity, in all matters relating to the reproductive system and to its functions and processes. Sexual health is concerned with the enhancement of life and personal relations, and not merely with counselling and care related to reproduction and sexually transmitted diseases.

Universal access to quality SRH information and services is essential to achieving the highest attainable standard for health, including SRH, and requires respecting, protecting and fulfilling the human rights of all individuals, including women living with HIV. In 2006, the World Health Organization (WHO) released the *Guideline on Sexual and Reproductive Health of Women Living with HIV/AIDS: Guidelines on Care, Treatment and Support for Women Living with HIV/AIDS and Their Children* [1]. In 2014, WHO advanced a framework which focused attention on specific human rights in the provision of contraceptive information and services [2]. Given the need to consider relevant rights with explicit attention to women living with HIV, along with changes in both the bio-medical and political aspects of the HIV response, a key step in support of advancing this process is a review of the literature to identify the existing evidence and gaps in interventions to address/promote human rights in the broader context of SRH programmes and policies for women living with HIV.

This literature review builds on a study by MacCarthy *et al*. [3] that examined the pregnancy-related needs, rights and decisions of women living with HIV and reported several issues: contraceptive options for pregnancy prevention for women living with HIV are insufficient – condoms are not always available or acceptable, and other options are limited by affordability, availability or efficacy; coerced sterilizations of women living with HIV is widely reported; information gaps persist in relation to effectiveness, safety and best practices regarding assisted reproductive technologies; attention to neonatal outcomes generally outweighs attention to the health of women before, during and after pregnancy; access to safe abortion and post-abortion care services are often curtailed; there is inadequate attention to HIV-positive sex workers, injecting drug users and adolescents; and the many challenges women living with HIV encounter in their interactions with SRH services contribute to their pregnancy decisions. Authors suggested it was critical that women living with HIV be more involved in the design and implementation of research, policies and programmes related to their pregnancy-related needs and rights. Authors noted several human rights issues in their analysis, including the availability, accessibility, affordability, acceptability and quality of services, participation, as well as the neglect of human rights more generally as requiring attention for the fulfilment of the SRH and rights of women living with HIV [3]. A review of United Nations and regional human rights norms and standards relevant to the SRH and human rights of women living with HIV are found elsewhere in this volume [4]. The objective of the present study is to review the current state of knowledge on the SRH and human rights of women living with HIV in order to assess evidence and gaps, and suggest areas requiring further study and action.

## Methods

With a focus on current knowledge, we conducted a targeted search of peer-reviewed and grey literature published in English, French or Spanish between July 1, 2011, and December 31, 2014. This review, which brings explicit attention to many aspects of the SRH and rights of women living with HIV, builds on the findings and conclusions of the literature review by MacCarthy *et al*. [3] summarizing the literature through June 2011 with an explicit focus on the pregnancy intentions of women living with HIV [3]. This review looks at the literature since that time and casts a wider net to include the topics included in the WHO guideline (2006) such as sexual health; family planning; antenatal, intrapartum, postpartum and newborn care; eliminating unsafe abortion; sexually transmitted infections (STIs), reproductive tract infections (RTIs) and cervical cancer, as well as violence and ageing. Databases including PubMed, Web of Science, Social Science Research Network (SSRN), Global Health, Public Affairs Information Service (PAIS) and International Bibliography of Social Sciences were searched for peer-reviewed literature using a combination of MeSH terms and keywords representing women, HIV/AIDS, ART, human rights and the above named areas within SRH. Databases including ProQuest Dissertations and Theses, New York Academy of Medicine Grey Literature Report, WHO Global Health Library, Scopus, Popline and PAIS were searched for grey literature using combinations of the keywords above. With respect to human rights, the decision was made to cast a wide net in order to include not only articles and documents where researchers had explicitly determined that human rights were part of their actions, but also those that were implicitly dealing with human rights using the nine agreed upon human rights dimensions found in the WHO Framework for Ensuring Human Rights in the Provision of Contraceptive Information and Services as the basis of this work [2] (i.e. equality; non-discrimination; participation; privacy and confidentiality; informed decision making; availability, accessibility, acceptability and quality (3AQ) of services individually or in their totality; and accountability) [2]. Thus, in order to capture the widest array of rights concepts to be found in the literature, search terms included also equity, stigma, respect and disrespect, as well as reproductive choice [2] (more details on the exact search strategy can be found in the appendix).

The peer-reviewed search results were imported into EndNote organized by SRH topic. By contrast, due to limitations in the search capacity of the grey databases, grey literature search results were imported into EndNote in relation to SRH more broadly. An initial review of each abstract or summary was completed followed by a full-text review of all articles and documents appearing to meet the inclusion criteria. Articles or documents were included in the review if they had as their primary focus the SRH and rights of women living with HIV or women receiving ART, including (but not limited to) SRH education and promotion, family planning, pregnancy, childbirth, eliminating unsafe abortion, STIs, violence and ageing. Finally, in order to broaden the pool of articles and documents reviewed, it was agreed that they need not focus only on women to be included, but that the extent to which attention was given to women in each specific case would be noted. Upon full-text review, any articles or documents which were then determined not to meet the inclusion criteria were removed.

Through this process, a final group of articles and documents meeting the inclusion criteria was identified. Of note, as there is a plethora of articles and documents that address stigma, but do not take discrimination or any other human rights standard into account in doing so, stigma-related articles and documents were included only if they explicitly made linkages to discrimination or other human rights. Relevant data (i.e. author(s), year, title, article/document type, abstract, population focus, geographic focus, human rights dimension(s) and SRH topic(s)) from all articles and documents identified for inclusion were abstracted and entered into an Excel table. Finally, a qualitative content analysis was conducted of the final pool of articles and documents to determine how exactly human rights had been addressed in each, including the types and frequency of mention of human rights and the context in which they were raised (i.e. human rights-based programming, human rights violations, etc.). Based on analysis and discussion of individual articles and documents within each of the SRH topics, broader themes that emerged within the topic were identified and are summarized in each topical sub-section of the results.

## Results and discussion

### Results of the search

**Figure 1:**
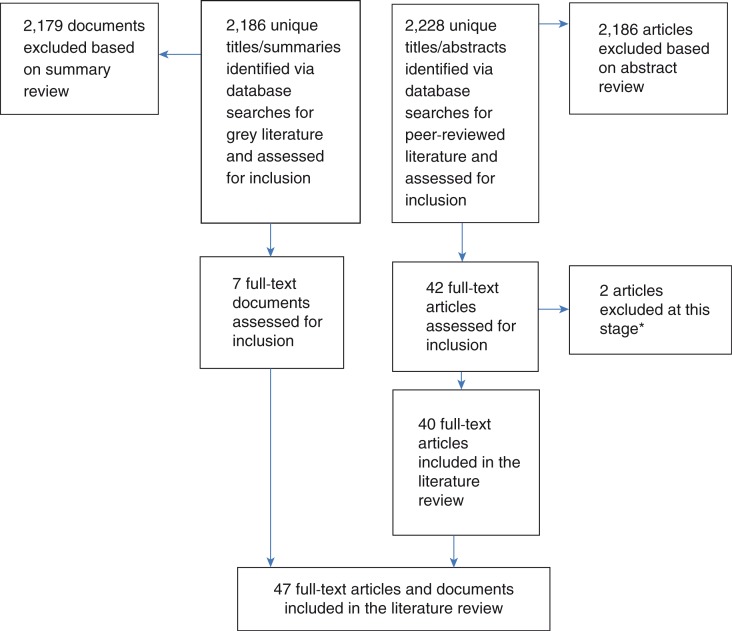
Results of the search strategy (peer-reviewed and grey). *Of the 42 full-text peer-reviewed articles assessed for inclusion, one was removed based on full-text review and one was removed due to inaccessibility of the full-text article.

The search of peer-reviewed literature yielded 2228 unique articles. All abstracts were reviewed and 42 were selected for full-text review, 40 of which met the inclusion criteria in the final review (Figure 1). Of the 40 publications, 32 represented original empirical studies (80% of findings) including 5 quantitative research studies, 14 qualitative research studies, 6 mixed methods studies and 7 policy analyses, while 8 (20% of findings) publications were either literature reviews, conceptual pieces, or viewpoints. Twenty-seven (68%) of these publications focused or drew on data from low- and middle-income countries (LMIC), while eight (20%) were global in scope. Thirty-nine (98%) of them were written in English, one in Spanish (2%) and none in French.

The search for grey literature yielded 2186 documents of which seven met the inclusion criteria in the final review. Of the seven documents, three were programme reports (43%), one was a programming guide (14%) and three were general documents related to the subject (43%). Three (43%) of these documents focused or drew on data from LMIC while four (57%) were global in scope. All seven (100%) of them were written in English.

Consistent with the topics included in the WHO guideline (2006), and the additional emphasis on violence and ageing, retrieved citations were organized by SRH topics pertinent to women living with HIV and women receiving ART. Upon review of the literature, it became evident the distinction between women living with HIV and those receiving ART was no longer pronounced in the literature. Results were therefore combined. In addition, the search brought to light some articles and documents which could not be easily placed within the identified topical areas but were relevant to the task at hand. These were ultimately placed in two new groupings: one on integrated models of care and the other on structural, societal and contextual factors impacting the SRH and rights of women living with HIV.

Table 1 indicates the final number of articles and documents that met the inclusion criteria according to the primary topic they addressed from the total citations retrieved.

**Table 1:** Number of articles and documents that met inclusion criteria by primary SRH topical area

Topics	Peer-reviewed articles: women living with HIV and/or women receiving ART (combined)	Grey documents: women living with HIV and/or women receiving ART (combined)
Promoting sexual health	3	1
Providing high-quality services for family planning	12	1
Improving antenatal, intrapartum, postpartum and newborn care	6	1
Eliminating unsafe abortion	5	0
Combating sexually transmitted infections, reproductive tract infections and cervical cancer	1	0
Reducing violence	1	2
Promoting healthy ageing	0	1
Integrated models of care	4	1
Structural, societal and contextual factors impacting sexual and reproductive health and rights	8	0
Total	40	7^a^
Total (peer-reviewed or grey)	47	

a Salamander Trust [8] is counted only once in the “promoting healthy ageing” category in the above table even as the document equally focused on other categories (i.e. promoting sexual health, providing high-quality services for family planning; and reducing violence) but is referred to in the text within each of those categories.

As the primary focus of this review was to determine how human rights concerns are addressed in the literature, an effort was made also to group articles so as to highlight which rights principles are most often addressed and with respect to which SRH topics. Table 2 presents all the peer-reviewed articles included in our review organized by author(s), year of publication, title, and the human right(s) and/or right(s)-related issue(s) addressed in each (i.e.(general) human rights, “human rights principles,” human rights violations, reproductive rights, reproductive and sexual rights, right to health, women's rights and rights-based approach). Table 3 presents all the grey documents in our review organized similarly by author(s), year of publication, title, and the human right(s) and/or right(s)-related issue(s) addressed in each.

**Table 2:** Human rights aspects addressed by peer-reviewed articles included in the review

Author(s)	Year	Title	Human right(s) and/or rights-related issue(s) addressed
C. Barroso & S. Sippel	2011	Sexual and reproductive health and rights: integration as a holistic and rights-based response to HIV/AIDS	Discrimination; equality; human rights violations; reproductive and sexual rights; rights-based approach; quality; women's rights
C. Beyrer, S. Baral, D. Kerrigan, N. El-Bassel, L.-G. Bekker and D. D. Celentano	2011	Expanding the space: inclusion of most-at-risk populations in HIV prevention, treatment, and care services	Accessibility; criminalization; discrimination; human rights principles; participation
C. H. Logie, L. James, W. Tharao and M. R. Loutfy	2011	HIV, gender, race, sexual orientation, and sex work: a qualitative study of intersectional stigma experienced by HIV-positive women in Ontario, Canada	Accessibility; discrimination
H. MacGregor and E. Mills	2011	Framing rights and responsibilities: accounts of women with a history of AIDS activism	Accessibility; gender inequality; privacy and confidentiality; reproductive and sexual rights; right to health
E. Mykhalovskiy	2011	The problem of “significant risk”: exploring the public health impact of criminalizing HIV non-disclosure	Criminalization; privacy and confidentiality
P. Orner, M. d. Bruyn and D. Cooper	2011	“It hurts, but I don't have a choice, I'm not working and I'm sick”: decisions and experiences regarding abortion of women living with HIV in Cape Town, South Africa	Discrimination; laws/policies; 3AQ
P. J. Orner, M. de Bruyn, R. M. Barbosa, H. Boonstra, J. Gatsi-Mallet and D. D. Cooper	2011	Access to safe abortion: building choices for women living with HIV and AIDS	Accessibility; laws/policies; reproductive and sexual rights
G. Anderson, G. Caswell, O. Edwards, A. Hsieh, B. Hull, C. Mallouris, … C. Nöstlinger	2012	Community voices: barriers and opportunities for programmes to successfully prevent vertical transmission of HIV identified through consultations among people living with HIV	Accessibility; human rights violations; participation; privacy and confidentiality; quality; reproductive and sexual rights
R. M. Barbosa, A. A. Pinho, N. S. Santos and W. V. Villela	2012	Exploring the relationship between induced abortion and HIV infection in Brazil	Accessibility; laws/policies; quality; reproductive and sexual rights
M. de Bruyn	2012	HIV, unwanted pregnancy and abortion – where is the human rights approach?	Accessibility; discrimination; human rights; laws/policies; reproductive rights; women's rights
O. Erhabor, C.I. Akani, & C.E. Eyindah	2012	Reproductive health options among HIV-infected persons in the low-income Niger Delta of Nigeria	Discrimination; laws/policies; reproductive and sexual rights; 3AQ
Z. Essack and A. Strode	2012	I feel like half a woman all the time: the impacts of coerced and forced sterilisations on HIV-positive women in South Africa	Discrimination; human rights violations; informed consent; reproductive and sexual rights
S. Fried, B. Harrison, K. Starcevich, C. Whitaker and T. O'Konek	2012	Integrating interventions on maternal mortality and morbidity and HIV: a human rights-based framework and approach	Accountability; discrimination; gender inequality; human rights violations; participation; right to health; 3AQ
E. Ghanotakis, D. Peacock and R. Wilcher	2012	The importance of addressing gender inequality in efforts to end vertical transmission of HIV	Accessibility; gender inequality
A. Gibbs, E. T. Crone, S. Willan and J. Mannell	2012	The inclusion of women, girls and gender equality in National Strategic Plans for HIV and AIDS in southern and eastern Africa	Accessibility; accountability; gender inequality; laws/policies; reproductive and sexual rights
A. Gibbs, M. Mushinga, E. T. Crone, S. Willan and J. Mannell	2012	How do national strategic plans for HIV and AIDS in southern and eastern Africa address gender-based violence? A women's rights perspective	Human rights violations; laws/policies; women's rights
C. H. Logie, L. James, W. Tharao and M. R. Loutfy	2012	We don't exist: a qualitative study of marginalization experienced by HIV-positive lesbian, bisexual, queer and transgender women in Toronto, Canada	Accessibility; discrimination; participation; quality
S. MacCarthy, J. J. K. Rasanathan, L. Ferguson and S. Gruskin	2012	The pregnancy decisions of HIV-positive women: the state of knowledge and way forward	Participation; reproductive and sexual rights; 3AQ
L. J. Messersmith, K. Semrau, T.L. Anh, N.N. Trang, D.M. Hoa, K. Eifler, & L. Sabin	2012	Women living with HIV in Vietnam: desire for children, use of sexual and reproductive health services, and advice from providers	Accessibility; discrimination; human rights violations; reproductive and sexual rights
G. Paz-Bailey, V. I. Fernandez, S. Morales Miranda, J. O. Jacobson, S. Mendoza, M. A. Paredes, D. C. Danaval, D. Mabey and E. Monterroso	2012	Unsafe sexual behaviours among HIV-positive men and women in Honduras: the role of discrimination, condom access, and gender	Accessibility; discrimination; gender inequality
M. M. Schaan, M. Taylor, J. Puvimanasinghe, L. Busang, K. Keapoletswe and R. Marlink	2012	Sexual and reproductive health needs of HIV-positive women in Botswana – a study of health care worker's views	Discrimination; 3AQ
J. A. Smit, K. Church, C. Milford, A. D. Harrison and M. E. Beksinska	2012	Key informant perspectives on policy- and service-level challenges and opportunities for delivering integrated sexual and reproductive health and HIV care in South Africa	Accessibility; laws/policies
A. Strode, S. Mthembu and Z. Essack	2012	She made up a choice for me: 22 HIV-positive women's experiences of involuntary sterilization in two South African provinces	Discrimination; informed consent; reproductive and sexual rights
C. J. Badul and A. Strode	2013	LM and Others v Government of the Republic of Namibia: the first sub-Saharan African case dealing with coerced sterilisations of HIV-positive women – Quo vadis?	Discrimination; human rights violations; informed consent
S. Bott and C. M. Obermeyer	2013	The social and gender context of HIV disclosure in sub-Saharan Africa: a review of policies and practices	Criminalization; human rights; laws/policies; privacy and confidentiality
B. Chimphamba Gombachika, E. Chirwa, A. Malata, J. Sundby and H. Fjeld	2013	Reproductive decisions of couples living with HIV in Malawi: what can we learn for future policy and research studies?	Accessibility; availability; laws/policies
K. Church, A. Wringe, P. Fakudze, J. Kikuvi, D. Simelane and S. H. Mayhew	2013	Are integrated HIV services less stigmatizing than stand-alone models of care? A comparative case study from Swaziland	Discrimination; 3AQ
G. G. Eamer and G. E. Randall	2013	Barriers to implementing WHO's exclusive breastfeeding policy for women living with HIV in sub-Saharan Africa: an exploration of ideas, interests and institutions	Accessibility; availability; laws/policies; participation
T. Kendall	2013	Falling short of universal access to reproductive health: unintended pregnancy and contraceptive use among Mexican women with HIV	Accessibility; discrimination; reproductive rights
E. J. King, S. Maman, J. M. Bowling, K. E. Moracco and V. Dudina	2013	The influence of stigma and discrimination on female sex workers’ access to HIV services in St. Petersburg, Russia	Accessibility; discrimination; quality
A. K. Laar	2013	Reproductive rights and options available to women infected with HIV in Ghana: perspectives of service providers from three Ghanaian health facilities	Quality; reproductive rights
M. Loutfy, U. Sonnenberg-Schwan, S. Margolese, & L. Sherr	2013	A review of reproductive health research, guidelines and related gaps for women living with HIV	Accessibility; discrimination; reproductive rights
M. Malta and C. Beyrer	2013	The HIV epidemic and human rights violations in Brazil	Discrimination; laws/policies; rights-based approach
K. Clouse, S. Schwartz, A. Van Rie, J. Bassett, N. Yende and A. Pettifor	2014	What they wanted was to give birth; nothing else: barriers to retention in option B plus HIV care among postpartum women in South Africa	Accessibility; discrimination; laws/policies
S. MacCarthy, J. J. K. Rasanathan, A. Crawford-Roberts, I. Dourado and S. Gruskin	2014	Contemplating abortion: HIV-positive women's decision to terminate pregnancy	Laws/policies; reproductive and sexual rights; 3AQ
P. Madhivanan, K. Krupp, V. Kulkarni, S. Kulkarni, N. Vaidya, R. Shaheen, … C. Fisher	2014	HIV testing among pregnant women living with HIV in India: are private healthcare providers routinely violating women's human rights?	Discrimination; human rights violations; informed consent; privacy and confidentiality; rights-based approach; right to health; quality
S. Malavé, J. Ramakrishna, E. Heylen, S. Bharat and M. L. Ekstrand	2014	Differences in testing, stigma, and perceived consequences of stigmatization among heterosexual men and women living with HIV in Bengaluru, India	Gender inequality
E. Marsicano, R. Dray-Spira, F. Lert, C. Aubriere, B. Spire and C. Hamelin	2014	Multiple discriminations experienced by people living with HIV in France: results from the ANRS-Vespa2 study	Discrimination
S. A. Spangler, M. Onono, E. A. Bukusi, C. R. Cohen, & J. M. Turan	2014	HIV-positive status disclosure and use of essential PMTCT and maternal health services in rural Kenya	Women's rights
M. G. van Dijk, K. S. Wilson, M. Silva, X. Contreras, H. D. Fukuda and S. G. Garcia	2014	Health care experiences of HIV-infected women with fertility desires in Mexico: a qualitative study	Discrimination; quality; reproductive rights

**Table 3:** Human rights aspects addressed by grey documents included in the review

Author(s)	Year	Title	Human right(s) and/or rights-related issue(s) addressed
Jain, S., Greene, M., Douglas, Z., Betron, M., & Fritz, K.	2011	Addressing HIV and gender from the ground up. Maanisha Community Focused Initiative to Control HIV: a program to build the capacity of civil society organizations in Kenya	Laws/policies; participation; women's rights
Khan, A.	2011	Gender-based violence and HIV: a program guide for integrating gender-based violence prevention and response in PEPFAR programs	Accessibility; discrimination; gender inequality; human rights; human rights violations; informed consent; participation; privacy and confidentiality; quality; women's rights
Kundu, N. K.	2011	SANGRAM's Collectives. Engaging communities in India to demand their rights	Discrimination; gender inequality; human rights; participation; rights-based approach
Open Society Institute	2011	Against her will: forced and coerced sterilization of women worldwide	Accessibility; accountability; discrimination; human rights violations; informed consent
Orza, L.	2011	Community innovation: achieving sexual and reproductive health and rights for women and girls through the HIV response	Accessibility; discrimination; gender inequality; human rights; laws/policies; participation; reproductive and sexual rights; rights-based approach; women's rights
Amnesty International	2014	Struggle for maternal health: barriers to antenatal care in South Africa	Discrimination; human rights violations; informed consent; privacy and confidentiality; right to health; 3AQ
Salamander Trust	2014	Building a safe house on firm ground: key findings from a global values and preferences survey regarding the sexual and reproductive health and human rights of women living with HIV	Discrimination; gender inequality; human rights; human rights principles; human rights violations; laws/policies; participation; reproductive and sexual rights; 3AQ

Findings are presented in the following sections mirroring the organization of the WHO guideline (2006), followed by the additional topics of violence; ageing; integrated models of care; and structural, societal and contextual factors. Within each category, findings are separated by those which emerged from the peer-reviewed literature, and those which emerged from the grey literature.

### Promoting sexual health

Of the 1469 citations that were retrieved from the peer-reviewed literature search in relation to promoting the sexual health of women living with HIV (as consistent with the WHO guideline this included SRH education, availability of information, access to family planning counselling, etc.), only three met the criteria for inclusion in this review. All three addressed disclosure of HIV status in one way or another. Taken together, several themes emerge: 1) there is a dearth of peer-reviewed literature on the promotion of sexual health for women living with HIV which includes attention to their rights, and what does exist is entirely within the context of disclosure; 2) research is needed as to how best to support women living with HIV in HIV disclosure not only in the immediate but throughout their sexual lives; and 3) discrimination by health providers based on HIV status for women who remain sexually active remains a persistent problem [5–7].

The search of the grey literature yielded a report describing a programme in India, SANGRAM Plus, which states that it deliberately implements rights-based principles in their approaches to enable collective transformation to help marginalized populations, including female sex workers living with HIV, to support one another, including increasing awareness of free health services available to people living with HIV and mechanisms to address violations. A second report from the grey literature presents findings from a global survey on SRH and human rights of women living with HIV. It represents the values and preferences of women living with HIV in a manner intended to be analogous to the form of building a safe house with foundations, walls, and a roof. Among the findings, it identifies challenges faced in a fulfilling and pleasurable sex life associated with fears of the consequences of disclosure, and difficulties with condom availability and negotiation. Taken together, these findings echo the need to support women living with HIV throughout their sexual lives and the findings also provide examples of a rights-based approach to programming to promote sexual health among this population [8,9].

### Providing high-quality services for family planning

Of the 149 citations that were retrieved from the peer-reviewed literature search in relation to family planning services for women living with HIV (as consistent with the WHO guideline this included family planning counselling, contraception and dual protection, sterilization, counselling for pregnancy planning, conception, etc.), 12 met the criteria for inclusion in this review. Taken together, the following key themes emerge: 1) the increased accessibility and availability of ART has profoundly impacted the ability of women living with HIV to realize their reproductive desires and rights although barriers still remain; 2) a wide range of experiences with healthcare providers has been reported and there is a demonstrated need to provide further clinical and rights-based training to providers to ensure women living with HIV can realize their reproductive desires and rights; 3) forced sterilizations continue to violate the rights of women living with HIV and must be addressed everywhere it occurs as a matter of urgency; and 4) recent court judgments are beginning to show promise in upholding the reproductive rights of women living with HIV although key barriers still remain [10–21].

The search of the grey literature found one report documenting cases of forced sterilization of women living with HIV in Chile, the Dominican Republic, Mexico, Namibia, South Africa and Venezuela [22]. The report on findings from a global survey on SRH and human rights of women living with HIV referenced earlier identifies a lack of quality care in family planning services for women living with HIV and calls for compassionate, holistic, unconditional care and support for informed choice [9]. Findings from these reports echo the demonstrated need to provide further rights-based training to providers to ensure women living with HIV can realize their reproductive desires and rights.

### Improving antenatal, intrapartum, postpartum and newborn care

Of the 233 citations that were retrieved from the peer-reviewed literature search in relation to antenatal, intrapartum, postpartum and newborn care (as consistent with the WHO guideline this included counselling during pregnancy, childbirth and the postpartum period; preventing HIV infection among infants; and skilled care during pregnancy, childbirth, and postpartum period), only six met the criteria for inclusion in this review. Taken together, the following themes emerge: while ART and related efforts have significantly reduced transmission of HIV from mothers to infants, 1) there remains inadequate attention to the challenges with disclosure and the attitudes of providers in the context of pregnancy, childbirth and postpartum care; and 2) despite some attention to human rights in prevention of mother-to-child transmission (PMTCT) programmes, more research is needed as to the potentially positive impacts of human rights-based approaches in PMTCT as well as other antenatal, intrapartum, postpartum and newborn care programmes [3,23–27].

The search of the grey literature yielded one report by Amnesty International in relation to antenatal care in South Africa which identified cases of discrimination and abuses by healthcare workers (including breaches of privacy and confidentiality) faced by women living with HIV. The findings in this report once again affirm that there remains inadequate attention to the discrimination faced by women living with HIV particularly in the context of pregnancy and childbirth [28].

### Eliminating unsafe abortion

While only three citations were retrieved from the peer-reviewed literature search in relation to abortion (as consistent with the WHO guideline this included abortion counselling, surgical and medical methods of abortion, post-abortion care and family planning), after full-text review of all articles meeting the inclusion criteria for review, five were ultimately found to be relevant to abortion. Taken together, the following key themes emerge: 1) restrictive abortion laws fundamentally infringe upon the reproductive rights of women living with HIV often resulting in the need to seek unsafe abortion; and 2) even in settings where abortion law is relatively liberal, there is a need for attention to other reasons that may result in women living with HIV needing to seek unsafe abortion including stigma and discrimination, socio-economic status and health service implementation factors [29–33].

The report on findings from a global survey on SRH and human rights of women living with HIV referenced earlier identifies a lack of informed consent in reproductive decision making. Findings suggest that more consistency is needed among global, regional and national policy and programming documents to ensure women living with HIV have access to safe abortion services and are able to exercise their reproductive rights [9].

### Combating STIs, RTIs and cervical cancer

Of the 252 citations that were found from the peer-reviewed literature search in relation to STIs, RTIs and cervical cancer (as consistent with the WHO guideline this included STIs and RTIs, screening for STIs, comprehensive case management, cervical cancer screening and treatment, etc.), only one article ultimately met the criteria for inclusion in the review. This study suggested the need to pay attention to rights in relation to reducing STI prevalence, improving service delivery and helping to establish an enabling legal and policy environment. No article made an explicit linkage between denial or promotion of rights and research findings. Overall, the limited literature on human rights and STIs, RTIs and cervical cancer affecting women living with HIV suggests the need for additional research with explicit attention to these linkages [34].

The search of the grey literature did not retrieve any documents specifically on this topic, echoing the need for additional research and documentation with explicit attention to these linkages.

### Reducing violence

Of the 53 citations that were retrieved from the peer-reviewed literature search in relation to violence among women living with HIV (as consistent with the WHO guideline this included sexual violence, physical assault and psychological violence), only one article had a primary focus on violence. This suggests an urgent need for research which considers the linkages between human rights; the various forms of violence, including gender-based violence, intimate partner violence, and structural forms of violence; and how these act as key barriers to HIV prevention, access, treatment and health outcomes for women living with HIV [35].

The search of the grey literature yielded a report of an initiative in Kenya which emphasizes the importance of community participation in addressing the links between HIV and gender-based violence [36]. In addition, the global survey report on SRH and human rights of women living with HIV referenced earlier identified the human rights dimensions of the violence experienced in homes, communities and institutions (including health services) and calls for safety in all of these settings [9]. Finally, the grey literature search yielded a guide for programme managers on integrating human rights into gender-based violence and HIV services [37]. While limited, these documents can offer insights into the need for and rights-based approaches to reduce violence among women living with HIV although more evidence is clearly needed in this area.

### Promoting healthy ageing

Of the 73 citations that were found from the peer-reviewed literature search in relation to ageing among women living with HIV, none gave attention to its human rights dimensions.

The search of the grey literature only retrieved the findings from the global survey on SRH and human rights of women living with HIV referenced earlier [9]. Respondents reported sizeable gaps in clinical care, practice, policy and research for women and girls outside the reproductive years and/or not wanting to have children and made a request for health services to adopt a holistic, women-centred con(tra)ception to old-age approach to sexual and reproductive healthcare, with a comprehensive package of age and stage-appropriate (i.e. infancy, adolescence, adulthood) services. This echoes the need for additional attention to human rights in relation to ageing for women living with HIV.

### Integrated models of care

Of the 40 articles included in this review from the peer-reviewed literature search, four emphasized integrated models of care as relevant to the SRH and rights of women living with HIV – three discussed integrated SRH and HIV services while another specifically discussed the potential synergies of integrating maternal health and HIV services. While some studies found clear consensus among decision makers on the need for more integrated systems of SRH and HIV care and the effectiveness integrated care can provide, a study from Swaziland suggested patients may experience increased felt-stigma in integrated settings. Taken together, the literature in this area suggests the following themes: 1) while integrated models of care likely provide opportunities to reduce discrimination and stigma for women living with HIV and improve accessibility and quality of care, results are not uniformly positive; and 2) rights-based approaches to SRH and HIV remain to be better defined but can elucidate both shared determinants and solutions in improving service delivery for and health outcomes of women living with HIV [38–41].

The search of the grey literature yielded one report addressing integrated models of care as relevant to the sexual and reproductive health and rights (SRHR) of women living with HIV. The report documents case studies of successful approaches at the intersection of SRHR and HIV innovated and/or led by women living with HIV with the intention of strengthening in practice the integration of services. Among the case studies are examples of projects integrating SRH and HIV services in the city of Memphis within the United States, programmes in Russia and Malawi reducing violence against women living with HIV, and home-based care for women and girls living with HIV in Kenya [42]. The report echoes that integrated models of care may improve accessibility and quality of care for women living with HIV although more research is needed.

### Structural, societal and contextual factors impacting SRH and rights

Of the 40 articles included in our review of the peer-reviewed literature, eight can be considered to have raised larger structural, societal and contextual factors impacting the SRH and rights of women living with HIV. Taken together, the literature in this area suggests the following themes: 1) gender inequality remains a key barrier to be addressed to improve the SRH of women living with HIV, with potential impacts also on all aspects of HIV prevention, access and treatment; and 2) discrimination against women living with HIV, and particularly women from key populations (in particular sex workers, injecting drug users and transgender women), continues to be pervasive and influence health behaviours, care-seeking, adherence to treatment and health outcomes [43–50].

Findings from all of the grey literature reviewed provide support and depth consistent with the conclusions above [8,9,22,28,36,37,42].

## Conclusions

Human rights increasingly form part of the language and approach of international organizations, governments, non-governmental organizations and civil society groups concerned with the SRH of women living with HIV. Most, if not all, States have committed themselves to promote and protect relevant human rights by ratifying international and/or regional human rights instruments, and making political commitments [51]. Beyond rhetorical commitments, the on-the-ground implications of a commitment to human rights for the lives and health of women living with HIV remain unclear. Even when efforts are made by States to improve the SRH of women living with HIV in line with their human rights commitments, legal, policy, structural and systems barriers continue to exist both inside and outside the health sector. Further, even as efforts are increasingly being made by a number of different actors to bring human rights into relevant SRH programming, documentation is inadequate and inconsistent. While there are tremendous strides taking place at the normative level and very important work happening on the ground, within the literature there are few well-documented examples of bringing human rights into the work undertaken to support the SRH of women living with HIV.

In this review, we found the amount of peer-reviewed literature to directly address human rights and the SRH of women living with HIV to be far more limited than expected in terms of quantity, and what does exist only addresses a few rights in the context of a few areas within SRH. Once the inclusion criteria were applied, the search yielded few articles. Even within the literature that met the criteria for inclusion, the language of rights is used most often to describe their apparent neglect or violation rather than their promotion or inclusion in programming or services. Several articles do point to the need to better integrate rights into interventions, particularly as it concerns provider training, raising client awareness and service delivery. From issues related to privacy and confidentiality to forced sterilization, awareness and integration of human rights in provider training is of critical importance. Increased education and information incorporating human rights is necessary for women living with HIV to claim their rights and make informed decisions regarding their healthcare. Finally, additional efforts must be made to test, validate and write up the inclusion of rights in comprehensive, quality care and service delivery models geared towards women living with HIV.

Similarly, the available and accessible grey literature in this search that directly addresses the application of human rights to address the SRH of women living with HIV is also limited. While a handful of examples were found, the grey literature search did not identify significant existing evidence on interventions to address/promote human rights in the context of SRH programmes and policies for women living with HIV. Rather, the grey literature offers significant examples of violations of rights and a few case studies of successful interventions. These give context and substance by providing information that demonstrates the SRH rights and needs of women living with HIV are real issues to be addressed.

While rights may be explicitly addressed in policies relevant to the lives of women living with HIV, there was not a single peer-reviewed article found through this review that described the conscious and explicit implementation of rights concepts in programming to address the SRH of women living with HIV. With the possible exception of one article by Fried *et al*. [40], none were found which addressed rights comprehensively, or addressed the majority of rights considered central to a rights-based approach, with only a handful addressing particular rights within a SRH topic. Several articles were included because it was determined that they met the criteria for implicit use of rights, particularly in relation to different aspects of the 3AQ, but it remains unclear if researchers had consciously brought rights concepts into these interventions, if they would as a consequence have been shaped differently and ultimately would have been more effective in health and rights terms. While it can be argued that articles and documents excluded from our review might also have raised rights implicitly to some degree, in particular the literature found on stigma, these lacked a sufficiently explicit link to human rights.

In sum, there appears to be a significant disconnect between those who are implementing rights-based interventions and those who are publishing in the peer-reviewed and grey literature. What can be found in the literature is limited, insufficiently funded and ultimately inadequate to represent the evidence base in this area. While it is possible that there may be wonderful examples of implementation of rights-based approaches to SRH of women living with HIV happening on the ground, this is not reflected in the literature, be it peer-reviewed or grey. There is an urgent need for more research in this area and to conduct rigorous interviews with institutions and organizations implementing rights-based interventions geared to supporting the SRH and rights of women living with HIV, including in-depth interviews with key informants working with women living with HIV (e.g. International Community of Women Living with HIV/AIDS (ICW), Global Coalition on Women and AIDS (GCWA), Athena, among others). Along with more research, attention to the normative work happening both globally and within regions and countries is warranted. Most importantly, the voices and experiences of women living with HIV must ultimately frame the discussion and inform evidence-based guidelines to improve the treatment, care and support of women living with HIV.
